# Case of Monstrosity

**Published:** 1854-06

**Authors:** D. M. Hudson

**Affiliations:** Bedford Co., Pa.


### *Case of Monstrosity.* By D. M. Hudson, M. D., Bedford Co., Pa.

(Communicated to Prof. F. G. Smith.)

Dear Sir,—A few days ago, in the course of my practice, I was called to see a new-born infant that came into the world without any eye-balls. After I had examined the child, I found it perfect in every other respect. The mother told me the following story: In the latter part of last August, while out with a friend gathering some whortle berries on some waste land, they came across a huge rattle-snake, which first bit their dog and then made battle with them. Much alarmed at this venomous reptile, the mother was seized with a nervousness which lasted during the rest of the day and greater part of the night. For two weeks the snake appeared before her eyes, and was uppermost in her mind, and at night when she awoke the creature was still present to her view. To get rid of this annoyance she rubbed her eyes until they were quite inflamed, and in this way she accounts for the absence of the eye-balls in her child. I questioned her as to the delivery of the child, if her midwife might not have injured the globes of its eyes. She said that nothing of the kind had taken place. I then asked if its eyes had suppurated much; she again answered in the negative, stating they were as I now saw them. On either side of the nose there is a small carbuncle, as it were, of a bluish gray cast, on the lower eyelids; these, she states, are the color of the snake. The child is a large male, and quite healthy. She gave birth to it on the 24th of April last.

I hope you will pardon me for the liberty I have taken in stating this case to you. Your opinion on it would afford me much gratification.

